# CO_2_ Desorption Performance from Imidazolium Ionic Liquids by Membrane Vacuum Regeneration Technology

**DOI:** 10.3390/membranes10090234

**Published:** 2020-09-14

**Authors:** Jose Manuel Vadillo, Lucia Gómez-Coma, Aurora Garea, Angel Irabien

**Affiliations:** Chemical and Biomolecular Engineering Department, E.T.S. de Ingenieros Industriales y Telecomunicación, Universidad de Cantabria, Avda Los Castros s/n, 39005 Santander, Spain; lucia.gomezcoma@unican.es (L.G.-C.); aurora.garea@unican.es (A.G.); angel.irabien@unican.es (A.I.)

**Keywords:** CO_2_ desorption, membrane vacuum regeneration, hollow fiber membrane contactor, Ionic liquid [emim][Ac], modeling

## Abstract

In this work, the membrane vacuum regeneration (MVR) process was considered as a promising technology for solvent regeneration in post-combustion CO_2_ capture and utilization (CCU) since high purity CO_2_ is needed for a technical valorization approach. First, a desorption test by MVR using polypropylene hollow fiber membrane contactor (PP-HFMC) was carried out in order to evaluate the behavior of physical and physico-chemical absorbents in terms of CO_2_ solubility and regeneration efficiency. The ionic liquid 1-ethyl-3-methylimidazolium acetate, [emim][Ac], was presented as a suitable alternative to conventional amine-based absorbents. Then, a rigorous two-dimensional mathematical model of the MVR process in a HFMC was developed based on a pseudo-steady-state to understand the influence of the solvent regeneration process in the absorption–desorption process. CO_2_ absorption–desorption experiments in PP-HFMC at different operating conditions for desorption, varying vacuum pressure and temperature, were used for model validation. Results showed that MVR efficiency increased from 3% at room temperature and 500 mbar to 95% at 310 K and 40 mbar vacuum. Moreover, model deviation studies were carried out using sensitivity analysis of Henry’s constant and pre-exponential factor of chemical interaction, thus as to contribute to the knowledge in further works.

## 1. Introduction

Carbon dioxide concentration in the atmosphere is continuing to increase due to the global energy demand, deeply dependent on fossil fuels, due to population and economic growth. Currently, the atmosphere value reaches 410 ppm, implying an increment of 50% with respect to the industrial revolution [1]. Thus, in recent years, different strategies have been widely studied to reduce CO_2_ emissions. On the one hand, promoting green energy sources, reducing the carbon-based fuel industry are calling attention. However, the use of energy with zero fuel fossil consumption is far from being completely implanted, and for this reason, it is necessary to find mechanisms to avoid CO_2_ emissions. Therefore, on the other hand, strategies are based on CO_2_ capture and sequestration (CCS), which is centered on CO_2_ long term storage, and CO_2_ capture and utilization (CCU) to convert CO_2_ into useful products [2]. Both types of strategies for <CO_2_ reduction require a first stage of carbon capture system, in accordance with the development of techno-economically sustainable technologies.

Traditionally, absorption columns (scrubbers) or packed beds working with aqueous amine solutions have been used for these separation processes, where the CO_2_ is absorbed into the amine solution by chemical reaction and then later desorbed by heating the CO_2_-rich solution [3]. The key challenges and, therefore, the main disadvantages to make efforts are the intensive use of energy for solvent regeneration and the associated environmental problems such as solvent loss and high volatility associated with direct gas-liquid contact [4]. Then, the motivation for the study of alternative processes is the reduction of energy consumption and the use of alternative solvents to the amine-based (beyond MEA), or several mixed solvents highly effective over long time periods.

CO_2_ separation using hollow fiber membrane contactors (HFMC) integrates the advantages of membrane separation and absorption, offering a determined interfacial area, independent control of gas and liquid flow rates, linear up-scaling, and avoidance of drop dragging [5]. However, one of the disadvantages of using membrane contactors is the mass transfer resistance, which increases if membrane wetting takes place [6]. In order to avoid wetting phenomena by the aqueous solution, hydrophobic microporous polymeric membranes, particularly membranes made of polypropylene (PP) and polytetrafluoroethylene (PTFE), have been extensively studied in recent years [7]. Despite of HFMCs not being CO_2_ selective, as the membrane does not provide selectivity to the separation since its role is to act as a barrier and to increase the surface for mass transfer exchange for both phases; the selection of the absorption solvent determines the selectivity of the separation. Moreover, in order to ensure the long-term application, and thus, the economic viability of CO_2_ capture, it is critical to have good compatibility between the solvent and the membrane contactor to avoid some issues related to the chemical resistance of the membrane as well as changes of polymer mechanical stability or swelling phenomena, increasing polymer size dilation [8,9].

Focusing on the selection of the absorbent as a key factor in preventing wetting, it is pointed out that PP membranes are not compatible with conventional amine solvents for extended contact times because of the chemical changes in the membrane surface structure and the low surface tension of the solvent [6]. Thus, the use of PP and PTFE hollow fiber membrane contactors require introducing alternative solvents in non-dispersive absorption. Several such amino acids and ammonia have been used in membrane contactors for the carbon capture system in the last years. Ammonia solution is a promising low-cost absorbent due to high CO_2_ loading capacity, the high chemical stability, and the lower regeneration cost as compared to conventional amine-based absorbents. However, the low CO_2_ reactivity and high volatility limited the economic and operational viability [10]. Amino acids have gained interest mainly because they have no environmental issues and because of their low volatility due to the ionic nature. However, amino acids solutions have some disadvantages as precipitation at high CO_2_ loadings, and high desorption energy requirement since the precipitated solvents must be heated up to re-dissolve the precipitates and regenerate carbon dioxide increasing the heat wasted [11].

Up to date, ionic liquids (ILs) are presented as promising absorbents alternative for conventional amine-based carbon capture, based on extensive reviews from many researchers [12,13]. ILs are organic molten salts with remarkable properties such as negligibly vapor pressure preventing solvent losses from volatilization in the gas stream, tunable structure, high thermal and chemical stability, low demand energy for regeneration, and excellent solvent power [14]. While they have been well studied for CO_2_ capture, ILs have several drawbacks that make their implementation into a gas capture system challenging. The high price of ILs is one of the limiting factors compared to conventional amine solvents. However, the lower cost of carbon capture using ILs can be achieved by decreasing manufacturing cost due to the increased demand and improving the efficiency in both capture and regeneration stages, decreasing the energy requirements. The price/performance ratio of ILs is then a key to compete with existing commercial solvents, taking into account that the tunability property of ILs can provide an extra degree of freedom for designing solvents [15]. Moreover, the high viscosity of the majority of ILs because of their ionic nature, leads to slow CO_2_ diffusion through the bulk IL, increasing the operational time requirements. However, this kinetic limitation may be addressed by providing a high surface area liquid-gas interface in the form of an HFM contactor [16].

Some review studies on CO_2_ absorption with HFMCs using ILs have been reported in recent years [17,18]. These studies have listed absorption capacities and parameters for a number of ILs with both physical and chemical absorption nature for CO_2,_ and the combination of ILs with membrane technology as a new approach for CO_2_ separation. Some previous papers that focused on this subject may also be referenced: Gomez-Coma et al. [19] studied the influence of temperature on physical and chemical absorption of CO_2_ with [emim][EtSO_4_] and [emim][Ac]; Albo and Irabien [20], performed the comparative analysis of CO_2_ capture in parallel-flow and cross-flow membrane contactors; Qazi et al. [21], described the CO_2_ absorption using various imidazolium ionic liquids as absorbents by a rigorous 2D mathematical model. However, studies on CO_2_ desorption via membrane contactors are relatively scarce, even though the desorption stage is responsible for the majority of energy consumption in PCC [22]. In order to ensure the stability of such membrane materials under long-term continuous operation, relatively low regeneration temperatures should be used; and to improve the regeneration rate, sweep gas operating mode, where inert gases like nitrogen or helium flow through the permeate phase (tube or shell) of the HFMC is typically used. Although using inert gas in sweeping mode is very useful to promote the CO_2_ mass transfer across the membrane, it would also bring an extra problem on how to enrich the CO_2_ stream for further CO_2_ valorization into value-added products. Therefore, vacuum-assisted CO_2_ stripping, where reduced pressure is applied by a vacuum pump to the permeate side, which may be an option to improve CO_2_ desorption performance without the purification process after CO_2_ capture.

CO_2_ Membrane Vacuum Regeneration (MVR) using hollow HFMCs is then presented as the most promising alternative allowing the process intensification. Regarding the intensification potential compared with conventional packed column strippers operating at high temperatures, MVR provides an equipment volume reduction of about 3 and a smaller solvent lost [23].

The results of the pilot and semi-commercial implementation projects of the absorption–desorption process of CO_2_ with the use of membrane contactors show some significant reduction percentages in weight and size characteristics of the equipment by 65–75%, capital costs by 35–40%, and operating costs by 38–42% [24]. However, to reach the industrial maturity and competitiveness of HFMC with packed columns, there is a need for viability tests under industrial conditions [25]. Up to date, this technology has been industrially evaluated using amines as absorbents. Wang et al. [26] studied CO_2_ MVR with 16 different amine-based absorbents showing better regeneration performance of the MVR process compared to the traditional thermal regeneration process at the same operating temperature. Fang et al. [27] reported that a decrease in the regeneration temperature required in MVR can contribute to reducing the solvent degradation rate. Nii et al. [28] showed that the MVR process can effectively utilize the low-temperature energy or waste heat in the power plants. However, since absorbent is selected based on properties such as CO_2_ selectivity and solubility, low regeneration energy, low volatility, and high contact angle with the membrane [29], it is necessary to carry out works based on ILs desorption using HFMCs.

Attempting to summarize the state of the art of the non-dispersive absorption/desorption of CO_2_ in HFMCs, the challenges that have to be faced in order to develop this technology in an industrial scale include wetting of the membrane (its implications to the mass transfer resistance and process efficiency), presence of other compounds in the gas stream (acting as competitors and limiting the mass transfer of the target compound), limited long-term stability (mainly related to the interaction of solvent and polymer, and the temperature effects), solvent issues (properties, costs; issues of concern from economic and environmental points of view that promote the use on non-volatile and tunable solvents such as ionic liquids), and solvent regeneration process since it determines the energy consumption significantly and thus the costs of the CO_2_ process capture.

Modeling and simulation issues include the challenges listed above for mass transfer and fluid flow accurate predictions, considering local variations, long time scales and wetting effects, scale-up predictions, and the systematic optimization of the membrane process to attain target performance, such as maximal process intensification and minimal energy requirements [7,8].

In addition, from the viewpoint of industrialization, recent progress on transport properties of IL fluids and process design, as well as the assessment of IL-based processes, were addressed in some reviews [17,30,31], covering relevant studies on CO_2_ capture and separation with conventional ILs, functionalized ILs, IL blending solvents, and IL-based membranes.

Considering these challenges and perspectives on the ionic-liquid-based CO_2_ capture systems, the motivation of this work is to contribute to the desorption process integration in the CO_2_ capture scheme with ILs, focusing on the study of the CO_2_ membrane vacuum regeneration process that has never been thoroughly investigated both experimentally and in modeling. A commercial polypropylene hollow fiber membrane contactor and two different commercial imidazolium-based ionic liquids as solvents were tested, 1-Ethyl-3-methylimidazolium ethyl sulfate [emim][EtSO_4_], which presents physical interaction with carbon dioxide and 1-ethyl-3-methylimidazolium acetate [emim][Ac], which also reacts chemically with CO_2_. The specific aims of this work are to provide new data of these imidazolium-based ILs related to the CO_2_ desorption behavior and to develop a comprehensive two dimensional (2D) mathematical model to study the CO_2_ membrane vacuum regeneration in a coupled system of a polypropylene HFMC and an IL. CO_2_ desorption tests were carried out thus as to compare MVR process performance using both loaded ILs. Experimental results of the absorption–desorption process operated at different operating conditions for desorption, with varying vacuum pressure and temperature used in order to model validation. CO_2_ desorption behavior and setup performance were studied in terms of MVR efficiency, CO_2_ flux, CO_2_ loading capacities, and module desorption profiles of CO_2_. Moreover, model deviation studies were carried out using sensitivity analysis of Henry’s constant and pre-exponential factor of chemical interaction.

## 2. Experimental Section

### 2.1. Materials

A commercial polypropylene hollow fiber membrane contactor (HFMC) (Liqui-Cel Membrane Contactor, USA) whose main specifications are listed in Table 1, was employed to both absorption and desorption stages. As absorbents, 2 different ionic liquids supplied by Sigma Aldrich were studied: 1-Ethyl-3-methylimidazolium ethylsulfate [emim][EtSO_4_] (≥95%) and 1-ethyl-3-methylimidazolium acetate [emim][Ac] (≥90%) were used. On the one hand, [emim][EtSO_4_] was selected due to its low viscosity, low cost, and the presence of only physical absorption [32]. [emim][Ac] was chosen as an absorbent because of its high CO_2_ solubility and results obtained in previous works, exhibiting both physical and chemical absorption. A gas mixture composed of 15% carbon dioxide (99.7, Air Liquide, Madrid, Spain) and 85% pure nitrogen (99.9%, Air Liquide, Madrid, Spain) was used as feed gas of the absorption stage.

The regeneration step was carried out using a condenser and a vacuum pump PC 3001 VARIO PRO purchased Vacuubrand. Control and measurement in the liquid line were carried out with a digital gear pump (Cole Parmer Instrument Company, Hucoa-Erloss SA, Madrid, Spain). The gas flow rate was measured and controlled by gas mass flowmeters (Alicat scientific, MC-gas mass flow controller, Madrid, Spain). Gas CO_2_ concentration was measured by a CO_2_ analyzer (Geotech, G110 0–100%, Coventry, UK).

### 2.2. Method

**Figure 1:**
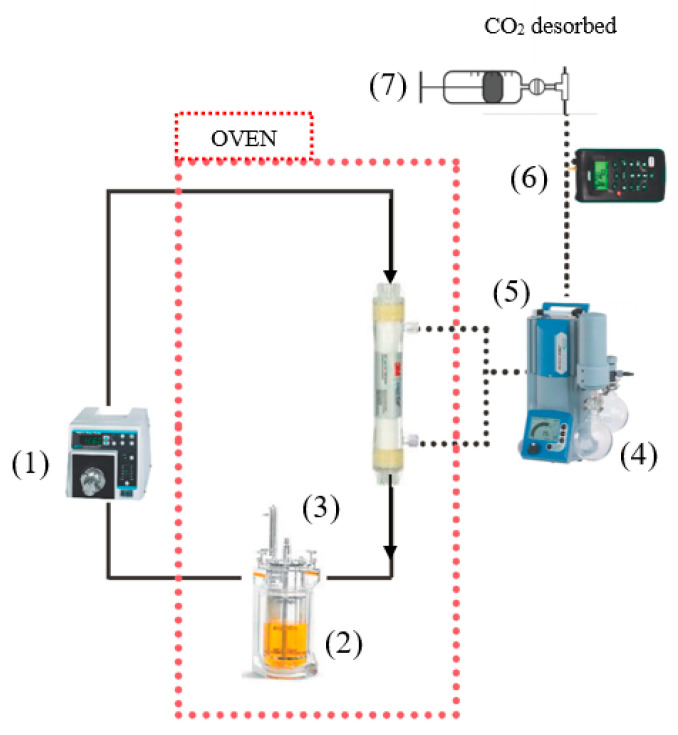
Experimental setup of the membrane vacuum regeneration (MVR) system. (1) Gear measuring pump; (2) solution tank with temperature control; (3) polypropylene (PP) HFMC; (4) condenser; (5) vacuum pump; (6) CO_2_ analyzer; (7) flow measurement by variable volume vessel.

A desorption test by MVR using a PP HFMC was carried out in order to determine the desorption or stripping capabilities of these CO_2_ loaded ILs for the further absorption–desorption process. Figure 1 shows the system set up for the configuration where the liquid flows through the tubes continuously while the vacuum is applied to the shell side.

Rich CO_2_ IL was continuously pumped into the tube side of the HFMC by the gear pump at 60 mL min^−1^. An experimental setup was kept inside an oven to maintain isothermal conditions during the MVR process. The shell side of HFMC was maintained at 40 mbar vacuum. The CO_2_ could be regenerated from the rich solution due to the positive effects of reduced pressure.

**Figure 2:**
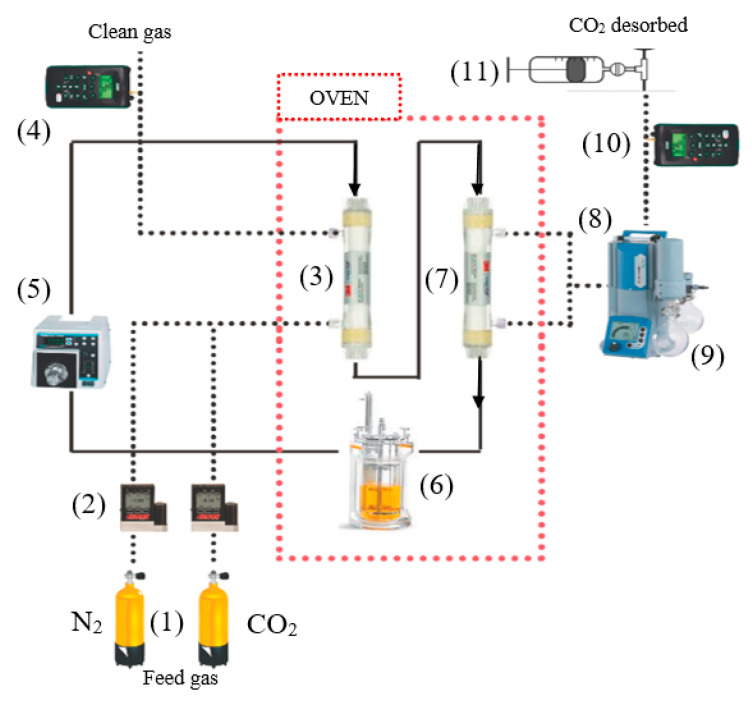
Experimental setup of the CO_2_ absorption–desorption process with one absorption HFMC and one desorption HFMC for MVR. (1) Gas cylinder; (2) mass flow controller; (3) absorption PP HFMC; (4) and (10) CO_2_ Analyzer; (5) gear measuring pump; (6) solution tank with temperature control; (7) desorption PP HFMC; (8) vacuum pump; (9) condenser; (11) flow measurement by variable volume vessel.

After the desorption tests by MVR were carried out, the experimental work with the CO_2_ absorption–regeneration process was accomplished. Figure 2 shows the experimental set up where both membrane modules were operating in the close loop system, under the conditions presented in Table 2.

The feed gas mixture contained CO_2_, 15% vol. and N_2_ (rest of balance), which was in the range of a typical coal-fired power plant (10–16%) [7]. The gas mixture (which flows in an open loop) was introduced through the shell side of the absorption module in counter-current at nearly atmospheric pressure. The IL was recirculated in a closed loop from a reservoir, through the fibers of the modules at a constant flow rate. IL was recirculated at a constant flow rate (60 mL·min^−1^), while the gas flow rate in the absorption stage was 60 mL·min^−1^. MVR was necessary to keep the shell side of the desorption module at reduced pressure. CO_2_ permeated from the rich solution to the shell side through the gas-filled membrane pores. Finally, the CO_2_ was enriched at the vacuum pump output.

Experiments were also performed inside an oven for a controlled temperature environment and isothermal conditions during the absorption–desorption process operation.

## 3. Model Development of MVR System in Absorption–Desorption Process

Most studies on the modeling of membrane contactors were focused on the CO_2_ absorption, and very limited research on the solvent regeneration process modeling has been carried out thus far, although the membrane contactors show potential in both direct CO_2_ stripping and integrated heat recovery [33].

The gas stripping membrane contactor can also be linearly scaled-up. A benefit of the membrane process is a straightforward scale-up because the available surface area between the gas and liquid phase is known; and as much higher values of gas-liquid interfacial areas can be provided compared to the scrubbing and stripping columns (typically 30 times), the size and costs of the units are then favored [34].

From this consideration, the HFMC modeling work was focused here on the CO_2_ stripping as follows.

### 3.1. Transport Equations

A 2D model was presented (for the desorption module set up in Figure 2) to study the mass transfer of CO_2_ in the MVR process using porous hydrophobic polypropylene HFMC. The rich CO_2_-[emim][Ac] was introduced into the tube side of the membrane contactor, while a vacuum was maintained on the shell side to reduce the partial pressure of CO_2_ in the gas phase. The CO_2_ component will be desorbed from the rich CO_2_-[emim][Ac] by penetrating the pores of the membrane from the tube side to the shell side. Due to the membrane hydrophobicity, the vacuum applied on the shell side was also applied in the membrane pores.

**Figure 3:**
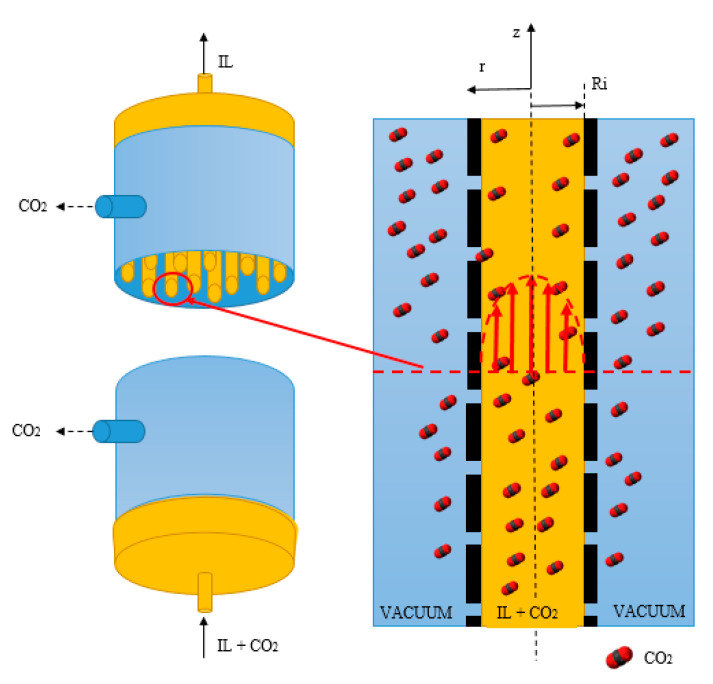
Diagram of CO_2_ MVR process in a hollow fiber membrane contactor.

In the mathematical model of the MVR process, the following assumptions were made: (1) Steady-state and isothermal conditions; (2) a fully developed parabolic laminar velocity profile was applied on the tube side; (3) negligible axial dispersion on the tube side; (4) physical and chemical absorption thermodynamic model applicable at the gas/liquid interface; (5) the [emim][Ac] concentration was kept constant throughout the process and (6) negligible pressure drop on the shell side. The schematic diagram of the CO_2_ transport in the desorption of CO_2_ in the membrane contactor using [emim][Ac] is shown in Figure 3.

**Figure 4:**
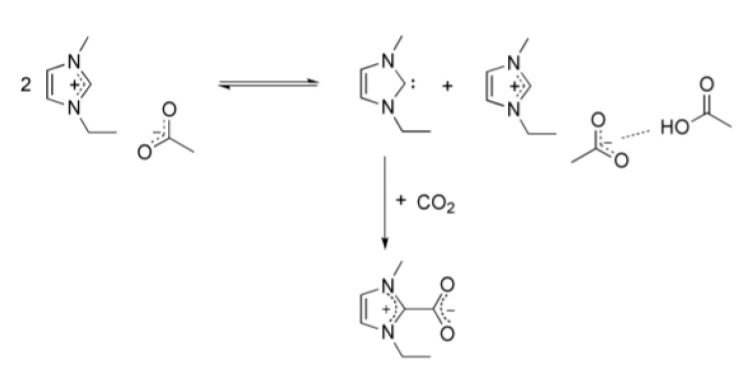
Proposed reaction of CO_2_ and [emim][Ac]. Reprinted with permission from Zareiekordshouli et al. [35]. Copyright (2018) Elsevier Ltd.

The desorption model takes into account that the concentration of CO_2_ in the liquid through the module was conditioned to the reversible chemical reaction [emim][Ac]-CO_2_ in the tube side of the module and to the mass transfer in the gas-liquid interface. The equation of steady-state continuity equations for the CO_2_ with simultaneous diffusion and chemical reaction within each fiber:

(1) $$V_{z}\frac{\partial C_{CO_{2}}}{\partial Z}=D_{CO_{2},l}\left(\frac{\partial^{2}C_{CO_{2}}}{\partial r^{2}}+\frac{1}{r}\frac{\partial C_{CO_{2}}}{\partial r}\right)-r_{CO_{2}},$$

where $V_{z}$ (m·s^−1^) is the liquid velocity in the axial direction; $C_{CO_{2}}$ (mol·m^−3^) is the physically dissolved CO_2_ concentration in the liquid side; $D_{CO_{2},l}$ (m^2^·s) is the CO_2_ diffusion coefficient in the liquid and $r_{CO_{2}}$ (mol·m^−3^·s^−1^) is the CO_2_ first order gas-liquid chemical reaction rate according to the reaction mechanism shown in Figure 4, which was calculated using Equation (2).

(2) $$r_{CO2}=k_{0}C_{CO_{2}}={B\;e}^{\left(\frac{-E_{a}}{RT}\right)}\;C_{CO_{2}},$$

where B is the pre-exponential factor of the chemical reaction equation (s^−1^) and $E_{a}$ is the activation energy (KJ·mol^−1^).

For its part, the velocity profile in the tube side can be determined with the following Newtonian laminar flow equation:

(3) $$V_{Z}=2V_{Zm}\left[1-{\left(\frac{r}{R_{i}}\right)}^{2}\right],$$

where $V_{Zm}$ (m·s^−1^) is the liquid mean velocity; and $R_{i}$ (m) is the membrane fiber inner radius.

The boundary conditions used to solve the model are expressed as follows

(4) $$C_{CO_{2}}=C_{CO_{2},0}\;\;\;\;\mathrm{for}\;Z=0,$$

(5) $$\frac{\partial C_{CO_{2}}}{\partial r}=0\;\;\;\mathrm{for}\;r=0,$$

(6) $$D_{CO_{2},l}\frac{\partial C_{CO_{2}}}{\partial r}=k_{ex}\left(C_{CO_{2},g}-\frac{C_{CO_{2},l}^{*}}{\frac{m}{E}}\right)\;\;\mathrm{for}\;r=Ri,$$

where $C_{CO_{2},0}$ is the physically dissolved CO_2_ concentration in the liquid phase at the initial time; $C_{CO_{2},g}$ is the gas phase CO_2_ concentration (mol·m^−3^); $C_{CO_{2},l}^{*}$ is the liquid phase CO_2_ concentration in the gas-liquid interface;m (-) is the distribution coefficient between the liquid and gas; E is the Enhancement factor; and $k_{ex}$ (m·s^−1^) is the combination of the mass transfer coefficients of CO_2_ in the membrane and in the gas phase;

(7) $${\left(k_{ex}\right)}^{-1}={\left(k_{g}\right)}^{-1}+{\left(k_{m}\right)}^{-1}.$$

It is assumed that the pores of the hydrophobic polypropylene membrane are filled with gas during the desorption and, therefore, the wetting membrane phenomena is negligible. The mass transfer coefficient of the membrane, $k_{m}$ (m·s^−1^), can be expressed as

(8) $$\frac{1}{k_{m}}=\frac{\tau \delta }{D_{CO_{2},m}\varsigma },$$

where ς (-) is the porosity; δ (m) is the membrane thickness; τ (m^−2^) is the tortuosity and $D_{CO_{2},m}$ (m^2^·s^−1^) is the effective membrane diffusion coefficient. As can be seen in Equation (8), higher porosity lead to lower mass transfer resistance from the tube side to the permeate side, while larger membrane thickness and tortuosity resulted in high mass transfer resistance by the membrane. However, a greater porosity level of the membrane might potentially result in a membrane with a higher wetting affinity, hence why a trade-off between porosity and wetting risk ought to be accounted for in order to secure and ensure not only the whole operation process, yet also its longevity and the high cost of the membrane.

On the other hand, the mass transfer coefficient in the gas phase ($k_{g}$) can be predicted by the following equation [36]:

(9) $$Sh=\frac{k_{g}d_{h}}{D_{CO_{2},g}}=5.85\left(1-\varphi \right)\left(\frac{d_{h}}{L}\right){Re}^{0.6}{Sc}^{0.33}\;\;\;\;0.04<\varphi <0.4,$$

where $D_{CO_{2},g}$ (m^2^·s^−1^) is the gas diffusion coefficient; φ is the packing density; L is the membrane fiber length; Re is the Reynolds, and Sc is the Schmidt, both dimensionless numbers; and; $d_{h}$ is the hydraulic diameter of the shell side:

(10) $$d_{h}=\frac{d_{\mathrm{cont}}^{2}-n\;d_{o}^{2}}{n\;d_{o}}.$$

The distribution factor (m), the relation between the CO_2_ concentration in the ionic liquid and the CO_2_ concentration in the gas phase, can be expressed by the following equation [37]

(11) $$m=\frac{\rho_{l}RT}{H_{CO_{2}}},$$

where $\rho_{l}$ is the molar density of the IL (mol·L^−1^); and $H_{CO_{2}}$ (MPa) is the Henry constant of CO_2_ in [emim][Ac] calculated in the Appendix A.

The enhancement factor (E) quantifies how the mass transfer is enhanced by the presence of a chemical reaction. In our work, E is predicted from experimental data by using a least-squares objective function and an optimization solver (NL2SOL) described in Section 3.3.

Finally, in order to compare both modeling and experimental results, the MVR cyclic efficiency ($R_{C}$) at pseudo stationary state was calculated as a function of cyclic absorption capacity $\alpha_{C}$ and CO_2_ loading of IL as

(12) $$\alpha_{C}=\alpha_{\mathrm{rich}}-\alpha_{\mathrm{lean}},$$

(13) $$R_{C}\left(\%\right)=\frac{\alpha_{\mathrm{rich}}-\alpha_{\mathrm{lean}}}{\alpha_{\mathrm{rich}}}\times 100,$$

where $\alpha_{\mathrm{rich}}$ is the CO_2_ loading in the IL (mol_CO_2_·mol_IL^−1^) before one pass of IL through the MVR module; and $\alpha_{\mathrm{lean}}$ is the CO_2_ loading in the IL (mol_CO_2_·mol_IL^−1^) reached in IL at the outlet of the MVR module calculated as dimensionless mixing cup (Equation (14)).

(14) $$\alpha_{\mathrm{lean}}=4\int_{0}^{\mathrm{Ri}}\alpha \left(1-r^{2}\right)r\;\mathrm{dr},$$

### 3.2. Physical Properties and Some Concerns of Model Parameters

Some physical properties required to solve the transport equations of the model. A representation of these values is listed in Table 3. Diffusivities, Henry constant, and distribution factors were calculated according to the Appendix A.

The Henry’s constant is an important parameter to be considered, which evaluates the intermolecular potential between a solute molecule and a solvent molecule. Henry’s law constant can be obtained conventionally from the experimental solubility data. For the case of chemical absorption for CO_2_ in ionic liquids, the conventional method becomes unreliable, large uncertainties may result with the blind use, depending strongly on the degree of the fitting polynomials, range of compositions, and whether the origin is included in the data set or not. Other (or often more rigorous) methods to obtain the Henry’s constant include the use of an Equation Of State (EOS) correlation for the entire experimental PTx data, as well as molecular simulation tools and some machine learning techniques for the estimation of the CO_2_ solubility as a function of the properties of ILs (w, Tc, Pc) and operating pressure and temperature [39,41].

The compilation of the solubility data of carbon dioxide in different ionic liquids, in the form of PTx curves [32,39,42] or expressed as molarity [43], show that many ILs do not obey a linear trend, and further analysis is needed to get a reliable value of Henry’s constant; being remarked the complex behavior in imidazolium acetate ionic liquids where chemical interaction is reported.

In our work, since [emim][Ac] was a physico-chemical solvent that reacts with CO_2_ to form a carboxylate and some conventional methods could not be reliable, the correlation developed recently by Hospital-Benito et al. [44] for carboxylate-based imidazolium ILs were used from the basis of ILs experimental isotherms fitted to a thermodynamic model. In this correlation, the physical absorption was described by Henry’s law and the chemical equilibrium reaction depending on the stoichiometry of reaction with CO_2_ depending on the IL [45].

### 3.3. Numerical Analysis

The modeling of the CO_2_ transferred from the liquid phase to the gas phase through the membrane was carried out using Aspen Custom Modeler (ACM) software as a numerical platform (Aspen Technology Inc., Bedford, Massachusetts, USA). For reasonable computing time, a single hollow fiber was considered for modeling.

The mass transfer equations and boundary conditions were related to the tube side. The radial and axial coordinates of the fiber are presented in Figure 3. Radial position r = 0 was pointed as the center of the fiber, and the axial distance of z = 0 represented the initial position of the gas in the fiber. The discretization method was based on the finite difference (FD) approach using the 4th order central finite difference (CFD4). Selecting an appropriate number of nodes (particularly in the radial direction) for the finite element analysis was very critical [46]. Under these considerations, 50 nodes were proposed in the axial and the radial directions to be in accordance with the 2-directional (r, z) mass transport.

Moreover, ACM software was used for the estimation task in order to predict the enhancement factor value. The optimal parameter was estimated by the minimization of an objective function. The combination of an objective function with an optimization solver was indicated as a solving method. In our work, the least-squares objective function and the solver NL2SOL were chosen. NL2SOL was an indirect solver as it calculated gradient information. The step choice algorithm was based on minimizing a local quadratic model of the sum of squares function constrained to an elliptical trust-region centered at the current approximate minimizer [47].

## 4. Results and Discussion

### 4.1. CO_2_ Desorption Test

**Figure 5:**
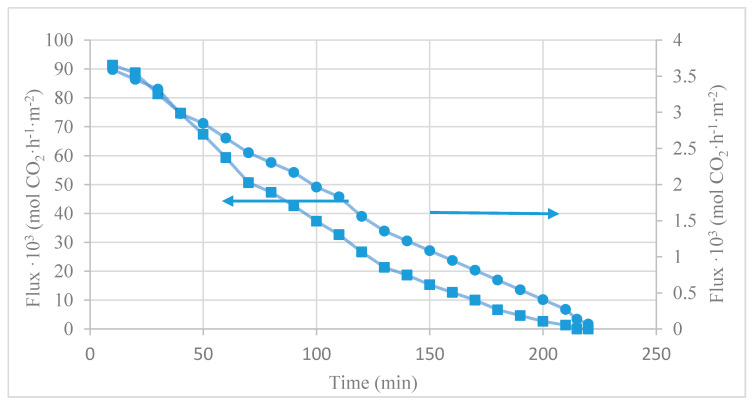
CO_2_ flux through the membrane vacuum regeneration (MVR) module over time in the CO_2_ desorption test, using different ionic liquids (ILs) being (●) [emim][EtSO_4_] and (■) [emim][Ac].

**Figure 6:**
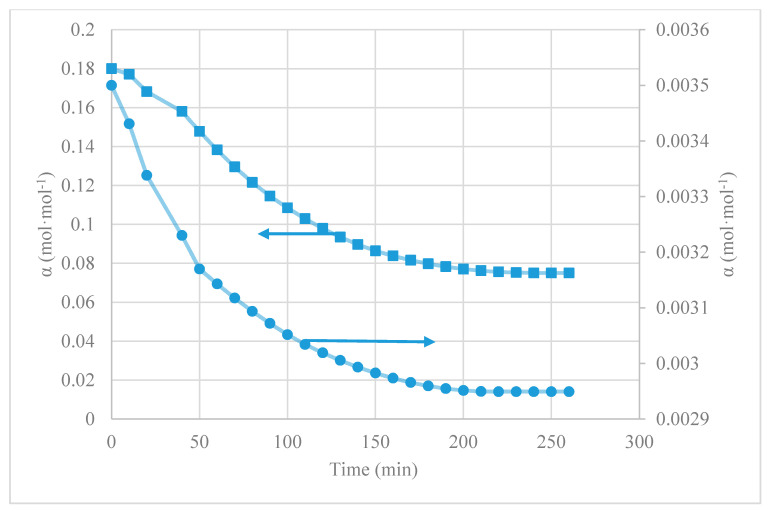
Change of CO_2_ loading of ILs with regeneration time in the CO_2_ desorption test, MVR process being (●) [emim][EtSO_4_] and (■) [emim][Ac].

The experimental set up of the desorption test was shown in Figure 1, Section 2.2. Experiments for the regeneration of rich CO_2_-loading ILs were performed in the MVR process. The rich CO_2_-loading ILs were obtained from the absorption operations. The CO_2_ loading change after regeneration Δα (mol_CO_2_·mol_IL^−1^) and the CO_2_ average desorption flux $N_{CO_{2}}$ (mol_CO_2_·h^−1^·m^−2^) were used to evaluate the regeneration performance in MVR using [emim][EtSO_4_] and [emim][Ac], as physical and physico-chemical absorbents, respectively. $N_{CO_{2}}$ can be expressed as

(15) $$N_{CO_{2}}=\frac{n\pi R_{i}^{2}C_{IL}V_{Z}\left(\alpha_{rich}-\alpha_{lean}\right)}{2n\pi R_{i}z}=\frac{R_{i}C_{IL}V_{Z}\Delta \alpha }{2z}$$

where $V_{Z}$ is the liquid velocity (m·h^−1^), n is the number of fibers, $R_{i}$ is the inner radius of the membrane (m), $C_{IL}$ is the concentration of fresh ionic liquid (mol·m^−3^), $\alpha_{rich}$ is the rich CO_2_-IL loading and $\alpha_{lean}$ is the CO_2_-lean average loading of IL in the output of the fiber. Figure 5 and Figure 6 show the CO_2_ desorption test results in terms of CO_2_ desorption flux and CO_2_ loading over time, respectively. The loading of the initial rich CO_2_-IL was 0.0035 mol_CO_2_·(mol_[emim][EtSO_4_])^−1^ and 0.18 mol_CO_2_·(mol_[emim][Ac])^−1^.

Experiments were carried out in a closed loop liquid flow mode in the operation conditions of room ambient temperature (289 K), vacuum pressure of 40 mbar, and liquid flow rate of 60 mL·min^−1^. Experiments were finished when CO_2_ concentration in the outlet of the vacuum pump reached zero.

Figure 5 shows the results of the instantaneous membrane fluxes, $N_{CO_{2}}$(t), of the MVR contactor coupled with both the physical absorbent [emim][EtSO_4_] and the physico-chemical absorbent [emim][Ac]. The obtained values of $N_{CO_{2}}$(t) with [emim][Ac] varied from 9.1·10^−2^ mol·m^−2^·h^−1^ to zero (220 min), while the $N_{CO_{2}}$(t) values corresponding to [emim][EtSO_4_] were reduced from 3.6 10^−3^ mol·m^−2^·h^−1^ to zero (in approximately 220 min experiment). The reason for this was that CO_2_ driving force decreased due to the decreases of CO_2_ loadings in the ILs with time during MVR, and thus reduced the $N_{CO_{2}}$(t). Since physico-chemical absorbents as [emim][Ac] reached higher CO_2_ loading after the absorption process ($\alpha_{rich}$) as other authors corroborate [32,48], it demonstrated that the desorption rate of the [emim][Ac] was much higher than that of the [emim][EtSO_4_].

Moreover, in order to bring more knowledge of desorption performance, experimental data regarding CO_2_ loading (α) versus regeneration time (t) is shown in Figure 6. Results indicate that the CO_2_ loadings of the ILs decreased with time by the membrane vacuum regeneration.

The CO_2_ loading of [emim][Ac] lowered with time and ultimately reached a constant 0.075 mol·mol^−1^, while [emim][EtSO_4_] approached a final value lower than 0.003 mol·mol^−1^. This is because [emim][Ac] requires to consume more energy to break the chemical bond due to CO_2_-[emim][Ac] chemical reaction and other regeneration methods as hot regeneration may be required to complete the regeneration on [emim][Ac]. Although, CO_2_ loading capacity and membrane flux using [emim][Ac] as a physico-chemical absorbent were much larger than that of the [emim][EtSO_4_] as a physical absorbent.

At this point, the membrane flux and CO_2_ loading versus regeneration time were studied for both physical and physico-chemical ILs absorbents. In order to further characterize the regeneration performances, MVR net efficiency ($R_{N}$) and net absorption capacity ($\alpha_{N}$) were presented for the regeneration process in Table 4. The difference between MVR cyclic efficiency (Equation (13)) and MVR net efficiency (Equation (17)) was that $R_{N}$ described the difference between CO_2_-rich solution and the final CO_2_ concentration reached when the close loop desorption process shown in Figure 1 reached a steady-state. While $R_{c}$ described the difference in CO_2_ concentration between input and output of the HFMC when the absorption–desorption process shown in Figure 2 reached a pseudo stationary state.

Moreover, MVR performances for different absorbents are obtained from literature to discuss the relationship. $R_{N}$ was calculated as

(16) $$\alpha_{N}=\alpha_{rich,0}-\alpha_{lean,t},$$

(17) $$R_{N}\left(\%\right)=\frac{\alpha_{rich,0}-\alpha_{lean,t}}{\alpha_{rich}}\times 100,$$

where $\alpha_{rich,0}$ is the CO_2_ loading in the IL at time zero; and $\alpha_{lean,t}$ is the CO_2_ loading in the IL reached when CO_2_ desorption test finished. The desorption test time (until steady-state) was 190, 220, 82, 56, 160, and 10 min for [emim][Ac], [emim][EtSO_4_], [bmim][BF_4_], [apmim][BF_4_], PG + AMP, and MEA, respectively.

Experimental results of MVR performance were analyzed as a function of MVR net efficiency and net absorption capacity. MVR efficiency can describe an absorbent as difficult or easy to be regenerated, while $\alpha_{N}$ represents the capacity of the absorbent to absorb CO_2_ after the MVR process. The results show that the chemical amino-based absorbents and physico-chemical ILs ([apmim][BF_4_] and [emim][Ac]) absorbent could not be fully regenerated, and the physical ILs ([bmim][BF_4_] and [emim][EtSO_4_]) could reach lower CO_2_ loadings after regeneration. It was pointed out that the $R_{N}$ using physical IL [emim][EtSO_4_] was 15%. The reason for this behavior may be attributed to the high viscosity that decreased the rich CO_2_ loading due to lower CO_2_ solubility in the IL.

For an absorbent selection, the $\alpha_{N}$ must be considered. In a practical absorption–desorption process, the volume requirement of an absorbent depends on the $\alpha_{N}$. An absorbent with a high $\alpha_{N}$ would result in small size equipment and low operation cost due to the low circulation volume required. Table 4 shows that the $\alpha_{N}$ of the physico-chemical absorbents are significantly higher than that of both physical ILs absorbents [bmim][BF_4_] and [emim][Ac]. As a result, in the absorption–desorption process for CO_2_ capture, performances of absorbents with chemical behavior may be better than that of the physical ILs.

The value of $R_{N}$ was 58% using [emim][Ac], while 34% was reached using a conventional MEA solution as absorbent. IL [emim][Ac] was considered as an efficient absorbent in the absorption process because the chemical interaction between CO_2_ and [emim][Ac], as other authors corroborate [38,42]. From the obtained results, it can also be pointed out that this IL is suitable for the CO_2_ desorption by MVR process.

### 4.2. Absorption–Desorption Process

**Figure 7:**
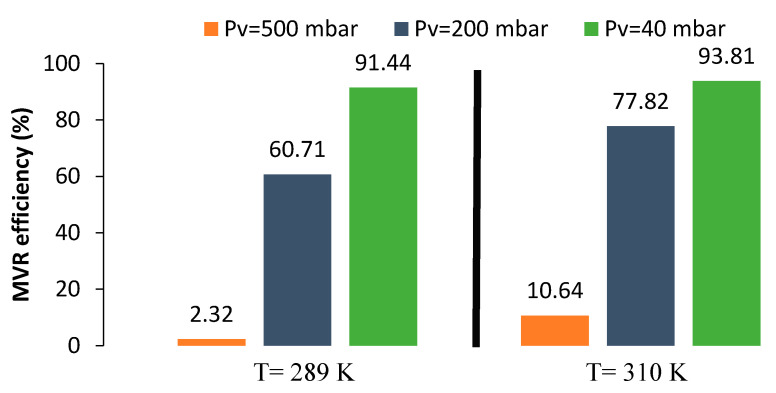
Effect of MVR vacuum pressure and temperature on desorption efficiency using [emim][Ac].

Experiments for the absorption–desorption process were carried out at different temperatures and vacuum pressures in order to evaluate MVR cyclic efficiency (Equation (13)) at different conditions after 200 min of recirculation (to ensure pseudo steady-state for all experiments). Although the liquid flow rate had a positive effect in the desorption process efficiency, it was kept constant within the ratio of liquid flow to gas flow (L/G) equals to one, due to the high viscosity of [emim][Ac] that increased the energy demand on the liquid pumping system, making such high values of L/G unfeasible. During recirculation of the absorption–desorption process shown in Figure 2, IL absorbs CO_2_ into the absorber, which leads to the accumulation of CO_2_ in the liquid side, part of which desorbs after entering the stripping module. The [emim][Ac] was recirculated until a constant CO_2_ concentration was achieved at the gas side outlet (reaching pseudo steady-state). Figure 7 shows the pseudo stationary efficiencies of MVR at different conditions.

**Figure 8:**
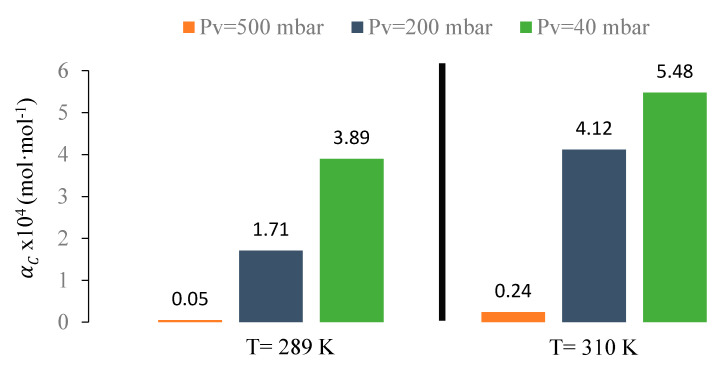
Effect of vacuum degree on the cyclic absorption capacity in a MVR process at different temperatures using [emim][Ac].

In order to better characterize the absorption–desorption process, the cyclic absorption capacity ($\alpha_{C}$) at different operational conditions was calculated, Figure 8. The cyclic absorption capacity calculated as $\alpha_{C}=\alpha_{rich}-\alpha_{lean}$ is the absorbent capacity to absorb CO_2_ after one cycle of the MVR process.

Figure 7 and Figure 8 compare both $R_{C}$ and $\alpha_{C}$ at different temperatures and vacuum pressures applied in the MVR process using [emim][Ac] as an absorbent. Higher desorption temperatures and vacuum pressures enhanced the desorption efficiency and thus reduced the specific energy consumption. Moreover, at higher $R_{C}$, higher cyclic absorption capacity corresponded due to the lower CO_2_ loading in the IL after one cycle of MVR. When experiments were carried out at room temperature (289 K), efficiencies of 3%, 60%, and 90% were achieved in contrast with 10%, 80%, and 95% working with 310 K at 500, 200, and 40 mbar, respectively. As previous works mentioned [19,35], higher temperatures imply more efficiency values. This can be attributed to temperature effects on both diffusion coefficients and CO_2_ solubility in the liquid phase. Koonaphapdeelert et al. [52] found that the mass transfer in gas stripping membrane contactors was mainly controlled by the liquid film mass transfer coefficient. Overall mass transfer coefficient using [emim][Ac] increased significantly as the temperature rose and vacuum pressure decreased: From 1.6·10^−6^ m·s^−1^ at 289 K and 500 mbar, to 3.2·10^−6^ m·s^−1^ at 310 K and 40 mbar, respectively. The values calculated in this work, based on the equation proposed by Gebremariam [53], were well within the range of values found in the literature for [emim][Ac] as an absorbent [38,54]. The mass transfer coefficient values calculated for ILs versus traditional amine-based absorbents were one order of magnitude lower, mainly due to their viscosity [48]. However, the use of ILs allows avoiding the main operational problems related to the amine-based solvents such as solvent volatility and solvent losses.

The overall mass transfer coefficient using [emim][Ac] is favored by temperature increases due to higher chemisorption phenomenon, lower viscosity, and higher diffusivity. For this reason, better performance is obtained operating at the highest possible temperature. Regarding membrane stability, the commercial polypropylene HFMC (Liqui-Cel) is unable to withstand temperatures more than 310 K due to their sensitivity to chemical and thermal attack. However, the hydrophobicity and low cost of commercial polypropylene modules could bring great advantage over other materials since the MVR process has the potential to reduce the regeneration temperature, which favors the reduction of steam costs in comparison to the thermal regeneration.

Moreover, lowering regeneration pressure (higher vacuum level) improves the efficiency by decreasing the partial pressure of CO_2_ in the gas phase of the MVR module, which would greatly favor the driving force of the mass transfer on the gas side of the HFMC; but also to imply the additional work for the vacuum pump and compression for downstream of membrane stripper. From these experiments, it is clear that pressure should be as low as possible. Regeneration pressure could be optimized at around a value in which the total equivalent work for MVR has the potential to be lower than thermal regeneration, and relative high regeneration efficiency could be satisfied. In consideration of low-grade steam is used in membrane vacuum regeneration, it will be more reasonable to compare energy consumption with thermal regeneration by total equivalent work. When the decreasing of regeneration pressure, overall equivalent work for CO_2_ membrane stripping decreases to a minimum value, being this variable subject to optimization. Compared with conventional thermal regeneration, up to 30% of energy consumption can be reduced for CO_2_ membrane vacuum stripping, as estimated by Wang et al. [55].

**Figure 9:**
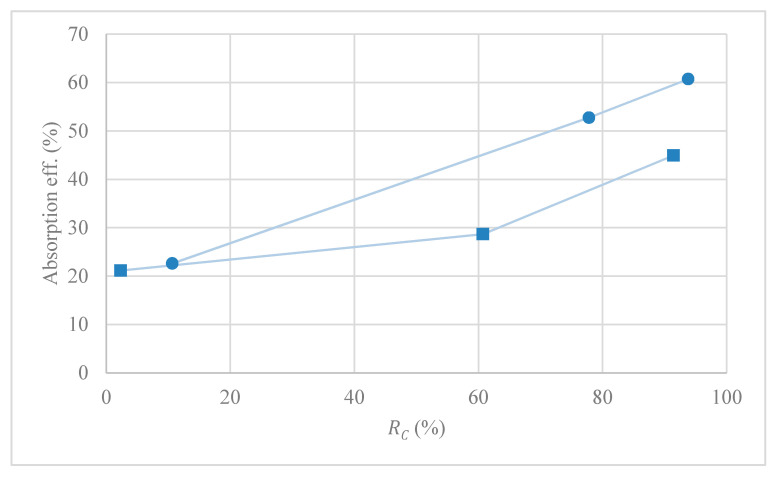
Correlation of CO_2_ absorption efficiency with MVR cyclic efficiency. [(■) Room temperature (289 K) and (●) 310 K].

Additionally, in order to show the influence of MVR module efficiency in the absorption–desorption process, steady-state absorption efficiency against MVR cyclic efficiency was studied in Figure 9. In this work, the efficiency of the absorption process in the pseudo-stationary state was calculated as,

(18) $$Absorption\;efficiency\left(\%\right)=\left(1-\frac{C_{CO2,g}^{out}}{C_{CO2,g}^{in}}\right)*100,$$

where $C_{CO2,g}^{out}$ is the CO_2_ concentration in the clean gas; and $C_{CO2,g}^{in}$ is the CO_2_ concentration in the feed gas.

**Figure 10:**
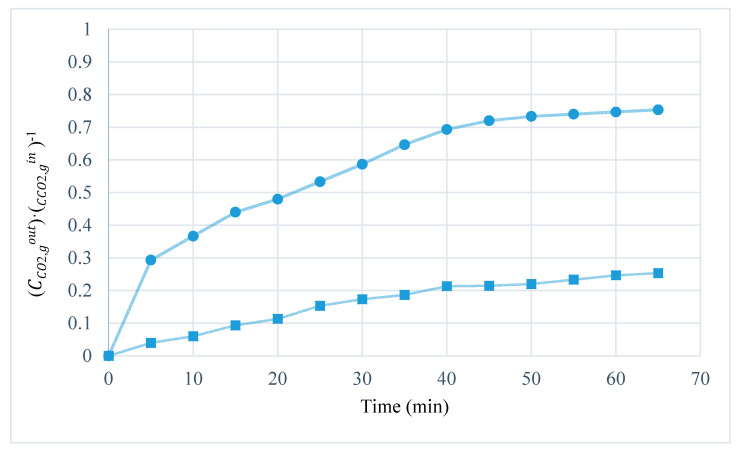
CO_2_ outlet concentration (dimensionless) vs. time. Influence of regeneration stage in absorption process. [(●) only absorption stage and (■) absorption–desorption process].

The maximum absorption efficiency (60%) was achieved when MVR cyclic efficiency was (93%), and cyclic absorption capacity reached the highest value (5.5·10^−4^ mol·mol^−1^). That was in accordance with Figure 8, with a higher cyclic absorption capacity and higher absorption efficiency due to the increase in the CO_2_ driving force in the absorption module. Performance of the absorption–desorption process has also been studied until a steady-state was reached in order to better understanding of the influence of the MVR process in the absorption process over time. Figure 10 shows the CO_2_ dimensionless average concentration $\left(\frac{C_{CO2,g}^{out}}{C_{CO2,g}^{in}}\right)$ over operation time for an absorption process using HFMC versus absorption–desorption process using MVR process at vacuum pressure (Pv) of 40 mbar and temperature of 310 K.

Previous studies [38] have reported efficiencies of 20–35% in the absorption process when [emim][Ac] was used as an absorbent. In this work, the efficiency in the pseudo-steady-state of the absorption process, which determined the CO_2_ capture efficiency, increased from 25% obtained in the absorption process to 74% in the absorption–desorption process (coupled system) using the best operating conditions (studied in this work) for the MVR process.

### 4.3. Model Validation

A robust model was developed using Aspen Custom Modeler and the results obtained were validated using the experimental data. Equations and model parameters were introduced in Section 3.

A Henry’s constant ($H_{CO_{2}}$) of 8.8 MPa was predicted using a correlation for chemical absorption between CO_2_ and imidazolium-based IL, proposed by Hospital-Benito et al. [44]. The average rate in the tube ($V_{Zm}$) was calculated by the formula described by Ghasem [36]. The diffusivity (D) was given by the diffusivity-viscosity correlation developed by Morgan et al. [56], and its dependence on temperature was assumed to be of the Arrhenius type. The term of the first order gas-liquid chemical reaction ($r_{CO_{2}}$) was estimated from the value of the overall mass transfer coefficient based on Qazi et al. [46].

An enhancement factor (E) of 62 was estimated from experimental data and model estimation tool. The E value is in agreement with previous data reported in the literature [19,57].

The concentration of CO_2_ in the gas side of the MVR module ($C_{CO2,g}^{v}$) was calculated from the vacuum pressure applied in the shell side of the MVR module (Pv) and the operation temperature.

**Figure 11:**
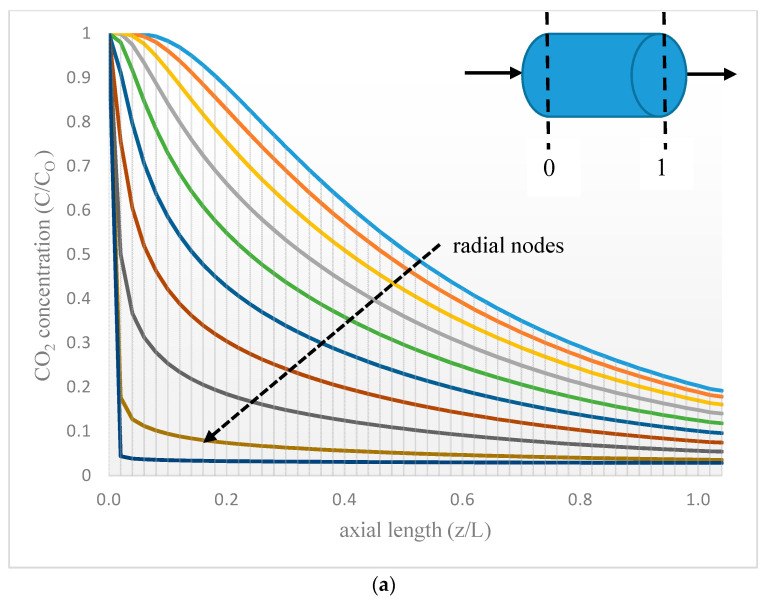

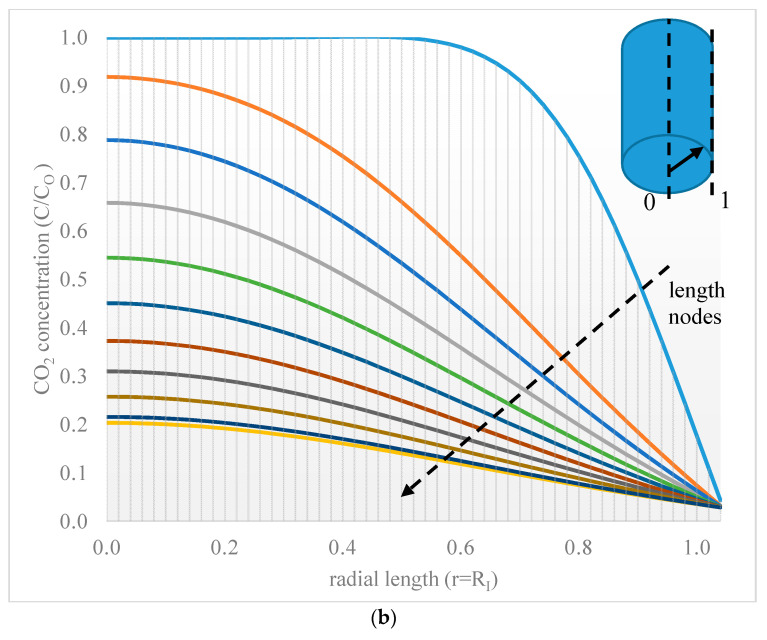
(**a**) CO_2_ dimensionless concentration in [emim][Ac] along fiber length. (**b**) CO_2_ dimensionless concentration in [emim][Ac] along fiber radius.

Figure 11 shows the modeling results of CO_2_ dimensionless concentration corresponding to the radial and axial dimensions for vacuum pressure of 40 mbar and room temperature in the hollow fiber module for desorption. Different nodes in the radial and axial dimensions are shown from the initial length (z = 0) to the final length (z = L) and from the center of the fiber (r = 0) to the membrane layer (r = $R_{i}$) (Figure 11a,b, respectively). The rich CO_2_-[emim][Ac] was introduced in the tube side from z = 0 to z = L, promoting a gradual decrease in CO_2_ concentration as it was desorbed progressively. The mass transfer CO_2_ profile was mainly determined by the driving force in the gas-liquid interface. Mass transfer in the radial direction is mostly affected by diffusion, while convection is the dominant mechanism for axial mass transfer due to liquid flow.

In order to validate the desorption model developed in this work, a comparison between experimental and simulated results of desorption efficiency was conducted, as shown in Table 5, from the different sets of operating parameters that were considered.

Model standard deviation error was calculated as:

(19) $$error\left(\%\right)=\frac{\left|experimental-model\right|}{experimental}*100,$$

The model predictions are in good agreement with the experimental result for different values of vacuum and temperature since the percent of variation explained by the fitting was higher than 95%. This agreement between both the model and experimental results shows the mathematical model flexibility towards variations in the MVR process parameters due to temperature and vacuum pressure changes.

Additionally, a sensitivity analysis of Henry’s constant ($H_{CO_{2}}$) of CO_2_ in [emim][Ac] (Equation (11)) and pre-exponential factor (B) of the first-order reaction rate constant (Equation (2)) were performed since these parameters were the ones with more uncertainty based on the literature. Just as an example, Henry’s constant is in the range of 0.0051 to 9.3 MPa because some studies do not take into account e tchemical equilibrium between CO_2_ and [emim][Ac] [39,40]. Our reference was the Henry constant calculated in our work ($H_{CO_{2}}$ = 8.8) by a correlation proposed by Hospital-Benito et al. [44], which was known to be very successful for the estimation of Henry constant for CO_2_ absorption in imidazolium ILs.

**Figure 12:**
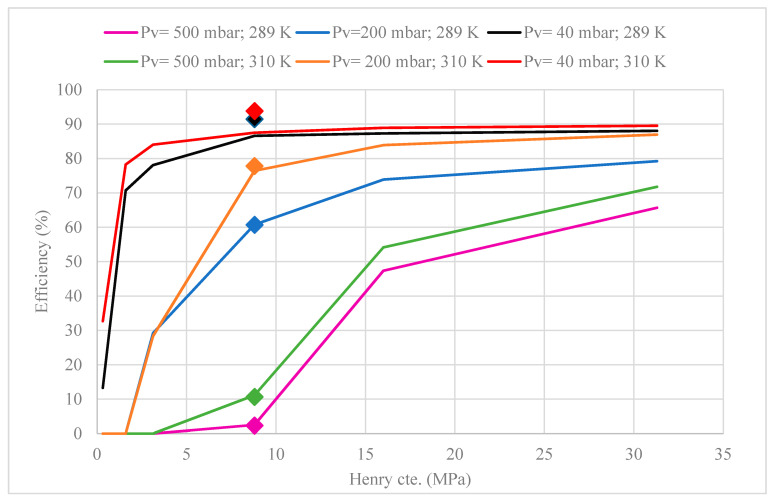
Sensitivity analysis of Henry constant in terms of MVR efficiency at different set parameters. [Legend: the scattered data points (♦) represent experimental data for each set of operating parameters].

Figure 12 shows the MVR efficiency predicted by the model at different Henry’s constant for the six sets of operating conditions used before. Higher Henry constant values increase MVR efficiency. This is because higher ($H_{CO_{2}}$) means that the absorbent is more able to absorb CO_2_ increasing the driving force in the gas-liquid interface. The sensitivity analysis indicated that at higher vacuum applied in the MVR system, which corresponds to higher desorption efficiencies, the Henry constant variability was less important, as the sensitivity of the model to this parameter was more critical when the desorption efficiencies were low.

**Figure 13:**
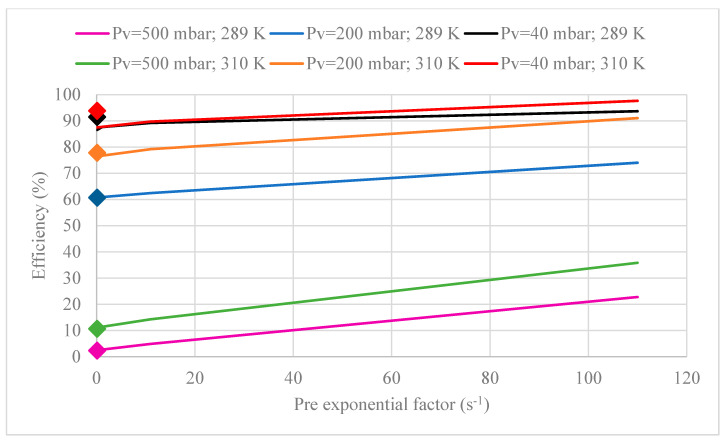
Sensitivity analysis of pre-exponential factor of the first-order reaction rate constant in terms of MVR efficiency at different set parameters. [Legend: the scattered data points (♦) represent experimental data for each set parameter].

Additionally, the pre-exponential factor value was reported in a range from 0.11 to 1545 s^−1^ since different approaches to estimate pre-exponential value were described in the literature [35,40]. However, Figure 13 shows that B value changes are slight in spite of the large range considered (from 0.11 s^−1^ to three orders of magnitude higher) because the mass transfer of CO_2_ in the proposed model mainly depends on the driving force in the gas-liquid interface.

## 5. Conclusions

The present work contributed to the desorption process integration in the CO_2_ capture and utilization scheme with ILs, focusing on the study of the CO_2_ membrane vacuum regeneration process using a polypropylene hollow fiber membrane contactor and two different commercial imidazolium-based ionic liquids as solvents. The approach for improving the desorption efficiency, which also implied improving the energy efficiency, is based on the application of lower vacuum pressures, as operation at a relatively low regeneration temperature is preferred, in conjunction with an intensified mass transfer equipment such a membrane contactor and an alternative solvent to the MEA solution.

Thus as to simulate the CO_2_ desorption from the IL in an MVR system through the use of a hydrophobic polypropylene HFMC, a two-dimensional (2D) mathematical model was developed. The experimental task was used to corroborate the model at diverse set parameter conditions of vacuum pressure (40, 200 and 500 mbar) and temperature (289 and 310 K). The modeling results of the MVR process were computed taking into consideration pseudo-steady-state in the absorption–desorption process, and the fitting was validated within a percent of variation explained higher than 95% related to the experimental behavior.

The IL [emim][Ac] as absorbent was chosen from a desorption test by MVR where [emim][EtSO_4_] and [emim][Ac] were tested in order to compare physical and physical-chemical absorbents by MVR net efficiency and net absorbent capacity.

In an absorption–desorption process, raising the temperature has a significant positive influence on the improvement of CO_2_ desorption performance. Along with this, more vacuum pressure applied to the MVR process enhances the CO_2_ desorption efficiency. Nevertheless, an increase of temperature and a decrease in regeneration pressure will lead to a rise in power cost, given a compromise between MVR performance and heat consumption.

A sensitivity analysis of Henry’s constant and pre exponential factor of the chemical reaction was developed in order to reduce uncertainty of the model due to the huge divergence of data available in the literature. Results showed the influence of the reaction constant was slight as the CO_2_ mass transfer was conditioned by the driving force in the gas-liquid interface, while further effort to the estimation of Henry’s constant is required for physical-chemical IL absorbents.

Taking into account the advances on the CO_2_ capture through the use of ILs in membrane contactors, it was remarked that the challenges for the application of the technology should cover the solvent regeneration process, since this process mainly determines the energy consumption and cost of post combustion carbon capture.

## Figures and Tables

**Table 1:** Hollow fiber membrane contactor (HFMC) characteristics (Liqui-Cel Membrane Contactor, Minneapolis, MN, USA).

Membrane Material	Polypropylene
Module i.d., d_cont_ (m)	25 × 10^−3^
Fiber outside diameter, d_o_ (m)	3 × 10^−4^
Fiber inside diameter, d_i_ (m)	2.2 × 10^−4^
Fiber length, L (m)	0.115
Number of fibers, n	2300
Effective inner membrane area, A (m^2^)	0.18
Membrane thickness, δ (m)	4 × 10^−5^
Membrane pore diameter, d_p_ (m)	4 × 10^−6^
Porosity, ς (%)	40
Packing factor, φ	0.39
Tortuosity, τ	2.50

**Table 2:** Operating conditions absorption–desorption process, laboratory scale.

Parameter/Property	Value	Unit
Ionic Liquid	[emim][Ac]	-
Volume, V	100	mL
Temperature, T	289–310	K
Feed Gas flow rate, Q_g_	60	mL·min^−1^
Liquid flow rate, Q_l_	60	mL·min^−1^
Feed gas pressure, P_g,in_	1.03	bar
Liquid pressure, P_l,in_	1.31	bar
Vacuum pressure, P_v_	0.04–0.5	bar

**Table 3:** Physical properties and mass transfer kinetics of [emim][Ac]-CO_2_ at 298 K.

Parameter	Unit	Value	Reference
Diffusion coefficient of CO_2_ in the liquid phase, $D_{CO_{2},l}$	m^2^·s^−1^	5.58·10^−10^	Appendix A
Diffusion coefficient of CO_2_ in the gas phase, $D_{CO_{2},g}$	m^2^·s^−1^	6.62·10^−6^	Appendix A
Diffusion coefficient of CO_2_ through the membrane, $D_{CO_{2},m}$	m^2^·s^−1^	1.86·10^−6^	Appendix A
Henry’s constant, $H_{CO_{2}}$	MPa	8.8	Appendix A
Liquid viscosity, $\mu_{l}$	cP	0.17	[38]
Liquid density, $\rho_{l}$	g·cm^−3^	1.1	[19]
Equilibrium constant, $K_{eq}$	-	136	[39]
Enthalpy, $∆H_{R}$	KJ·mol^−1^	−30.18	[40]
Activation energy, $E_{A}$	KJ·mol^−1^	9.2	[40]

**Table 4:** Regeneration performances of various absorbents in a CO_2_ desorption test by MVR system using polypropylene hollow fiber membrane contactors (PP HFMCs).

Solvent	$\alpha_{\mathrm{rich}}$	$\alpha_{\mathrm{lean}}$	$\alpha_{N}$	$R_{N}\left(\%\right)$	Reference	Operational Conditions
[emim][Ac]	0.180	0.075	0.105	58	our work	Room T; 40 mbar
[emim][EtSO_4_]	0.0035	0.003	0.001	15	our work	Room T; 40 mbar
Aqueous [bmim][BF_4_]	0.018	0.000	0.018	100	[49]	Room T; 500 mbar
Aqueous [apmim][BF_4_]	0.380	0.150	0.230	61	[49]	Room T; 500 mbar
Aqueous PG + AMP	0.900	0.550	0.350	39	[50]	Room T; 600 mbar
Aqueous MEA	0.69	0.454	0.236	34	[51]	70 °C; 100 mbar

**Table 5:** Experimental and model desorption efficiency for different set conditions.

			Experimental	Model	Error.
SET	Pv (mbar)	T (K)	$R_{C}$ (%)	$R_{C}$ (%)	(%)
1	500	289	2.3	2.5	7.3
2	200	289	60.7	60.7	0.1
3	40	289	91.4	86.6	5.3
4	500	310	10.6	11.1	4.5
5	200	310	77.8	76.4	1.8
6	40	310	93.8	87.5	6.7

## Abbreviations

C	molar concentration, mol CO_2_·mL^−1^
D	diffusion coefficient, m^2^·s^−1^
$d_{cont}$	diameter of the contactor, m
$d_{h}$	hydraulic diameter of the shell side, m
$d_{i}$	inside diameter of the fiber, m
$d_{o}$	outside diameter of the fiber, m
E	enhancement factor
Ea	activation energy, KJ·mol^−1^
H	Henry’s constant, MPa
$∆H_{R}$	enthalpy, KJ·mol^−1^
$k_{o}$	first order reaction rate constant, s^−1^
$K_{eq}$	equilibrium constant
k	mass transfer coefficient, m·s^−1^
m	distribution coefficient between the liquid and gas
M	molecular weight, g·mol^−1^
N	desorption molar flux, mol·m^−2^·h^−1^
P	pressure, bar
$P_{V}$	vacuum pressure applied in MVR process, bar
Q	volumetric flow rate, mL min^−1^
R	MVR efficiency, %
$R_{i}$	inner radius of membrane fiber, m
r	radial coordinate
$r_{CO_{2}}$	CO_2_ reaction rate, mol·m^−3^·s^−1^
T	temperature, K
V	volume, mL
$V_{z}$	velocity, m·s^−1^
$V_{zm}$	liquid mean velocity, m·s^−1^
$V_{CO_{2}}$	molar volume of CO_2_, cm^3^·mol^−1^
z	axial coordinate
Re	Reynolds number
Sc	Schmidt number
Sh	Sherwood number
**Subscripts**	
$CO_{2}$	carbon dioxide
C	cyclic
ex	external
0	initial
g	gas
IL	ionic liquid
*in*	inlet
l	liquid
m	membrane
N	net
out	outlet
v	vacuum
**Greek Letters**	
α	CO_2_ loading, mol_CO_2_·mol_IL^−1^
μ	viscosity, cP
ρ	density, Kg·m^−3^
υ	molar volume of CO_2_, cm^3^·mol^−1^

## Appendix A

### Appendix A.1. Liquid Diffusivity

Morgan et al. developed a correlation, which expresses the dependency of gas diffusivity with the liquid viscosity as [56]:

(A1) $$D_{CO_{2},l}=3.7\cdot 10^{-3}\frac{1}{\mu_{IL}^{0.59\pm 0.02}\upsilon_{CO_{2}}^{1.00\pm 0.07}\rho_{IL}^{2.0\pm 0.1}},$$

where $\mu_{IL}$ is the viscosity of IL in cP; $\rho_{IL}$ is the IL density in g·mL^−1^; $\upsilon_{CO_{2}}$ is the molar volume of carbon dioxide at the normal boiling point (33.3 cm^3^·mol^−1^) and the diffusivity is obtained in cm^2^·s^−1^. Assuming an Arrhenius-type dependence of temperature, the diffusivity is calculated from room temperature to 318 K [38].

### Appendix A.2. Gas and Membrane Diffusivities

The CO_2_ diffusion coefficient in the gas phase can be calculated by the Maxwell–Gilliland equation [58]:

(A2) $$D_{CO_{2},g}=\frac{4.36\cdot 10^{-5}T_{G}^{3/2}\sqrt{\frac{1}{M_{CO_{2}}}}}{P\upsilon_{CO_{2}}^{1/3}},$$

where $T_{G}$ is the gas-phase temperature; $M_{CO_{2}}$ is the molecular weight of CO_2_ (in g/mol) and P is the pressure in the gas phase (kPa).

A porous membrane is used in this work for which the effective diffusivity is found by the following equation [46]:

(A3) $$D_{CO_{2},m}=D_{CO_{2},g}\frac{\varepsilon }{\tau },$$

where *ε* and *τ* are porosity and tortuosity of the porous membrane.

### Appendix A.3. Henry Constant

The Henry constant commonly adjust the ILs experimental isotherms performed by Shiflett and Yokozeki [41] to a thermodynamic model in which the physical absorption is described by Henry’s Law. In our work, chemical absorption is taken into account and is described by the chemical equilibrium reaction depending on the stoichiometry. Reaction between CO_2_ and [emim][Ac] is described as 1:2 stoichiometry (2 [emim][Ac] + CO_2_ ↔ PROD):

(A4) $$z=\frac{P_{CO_{2}}}{K_{H}-P_{CO_{2}}}+\frac{-2K_{eq}\frac{P_{CO_{2}}}{K_{H}}+\sqrt{K_{eq}\frac{P_{CO_{2}}}{K_{H}}}}{1-4K_{eq}\frac{P_{CO_{2}}}{K_{H}}},$$

where z is the molar ratio of CO_2_ absorbed per mol of IL, $P_{CO_{2}}$ is the CO_2_ partial pressure in bar, $K_{H}$ is the CO_2_ Henry’s law constant in the IL in bar, $K_{eq}$ is the reaction equilibrium constant.

The chemical CO_2_ absorption is related to the obtained reaction equilibrium constant ($K_{eq}$) and heat of reaction (Δ*HR*). Slightly larger CO_2_ solubility may be expected if compared to Henry’s law constants calculated for CO_2_ in ILs that only present physical absorption [59].
